# The Maximum Entropy Fallacy Redux?

**DOI:** 10.1371/journal.pcbi.1004777

**Published:** 2016-05-12

**Authors:** Erik Aurell

**Affiliations:** 1 Department of Computational Biology, ACCESS Linnaeus Centre and Centre for Quantum Materials, KTH-Royal Institute of Technology, Stockholm, Sweden; 2 Departments of Information and Computer Science and Applied Physics, Aalto University, Espoo, Finland; Politecnico di Torino, ITALY

Protein structure can be predicted in silico given sufficiently good templates, as demonstrated in successive installments of the biannual Critical Assessment of protein Structure Prediction (CASP) competition [1]. Since the number of known protein sequences currently increases much faster than the number of known protein structures, and is likely to continue to do so in the foreseeable future, reliable ab initio protein structure prediction, without recourse to templates, would be highly desirable. It is currently not possible to achieve sufficient sampling in unrestrained folding to achieve predictions close to the native structure. Recently, very important progress has been made on the restricted problem of predicting spatial amino acid contacts in proteins from many homologous sequences [2]. While it is not yet clear if these techniques, collectively known as direct coupling analysis (DCA), can be leveraged to systematically predict full protein structures, preliminary results indicate that this may be the case [3,4]. DCA has also been used with success for several related problems, such as predicting structures of protein complexes [4] or alternative protein conformations [5].

The central ingredient in DCA is to learn generative probabilistic models from a set of homologous protein sequences. These models are chosen from an exponential family with linear and quadratic interactions, commonly referred to as Potts models (see Eq 1) [6]. In the literature, this procedure has been motivated by maximum entropy arguments [7–10]. In this Perspective, I will point out that these arguments are mistaken and that the successes of DCA can have nothing to do with maximum entropy. To the contrary, maximum entropy hides the real nature and questions raised by DCA and is thus an obstacle to progress. In addition, maximum entropy has a long and contested history in statistical physics, the field in which it was first introduced [11,12]. Definite and precise results derived in the last decade and a half have here conclusively falsified maximum entropy. Appeals to maximum entropy are therefore prejudicial to a more general acceptance and adoption of DCA.

## Maximum Entropy and DCA

Maximum entropy was introduced by E.T. Jaynes in two papers published in *Physical Review* in 1957 as an alternative view of both equilibrium and nonequilibrium statistical physics [11,12]. The most concise statement can be found in the second of these two papers, and reads, “the probability distribution over microscopic states which has maximum entropy, subject to whatever is known, provides the most unbiased representation of our knowledge of the system.”

For a positive evaluation of maximum entropy in science and an entry point to the more recent literature, I refer to the companion paper by Erik van Nimwegen [13].

The maximum entropy argument for DCA starts from a multiple sequence alignment of homologous protein sequences and posits that each sequence is an independent draw from a probability distribution maximizing entropy while constrained by the single-site amino acid frequencies *f*_*i*_(*k*) and two-site amino acid pair frequencies *f*_*ij*_(*k*,*l*). In these two characteristics, *i* and *j* range over the length of the alignment and *k* and *l* range from 1 to 21, where states 1–20 are the naturally occurring amino acids and state 21 is a gap state. By a short calculation, essentially found in [14], the probability distribution then takes the exponential form, with linear (*h*_*i*_(*a*_*i*_)) and quadratic (*J*_*ij*_(*a*_*i*_, *a*_*j*_)) interactions and a normalization constant *Z*

$$P(a_{1},\ldots ,a_{N})=\frac{1}{Z}\text{exp}\left(\underset{i}{\sum }h_{i}(a_{i})+\underset{ij}{\sum }J_{ij}(a_{i},a_{j})\right)$$ (1)

**Fig 1:**
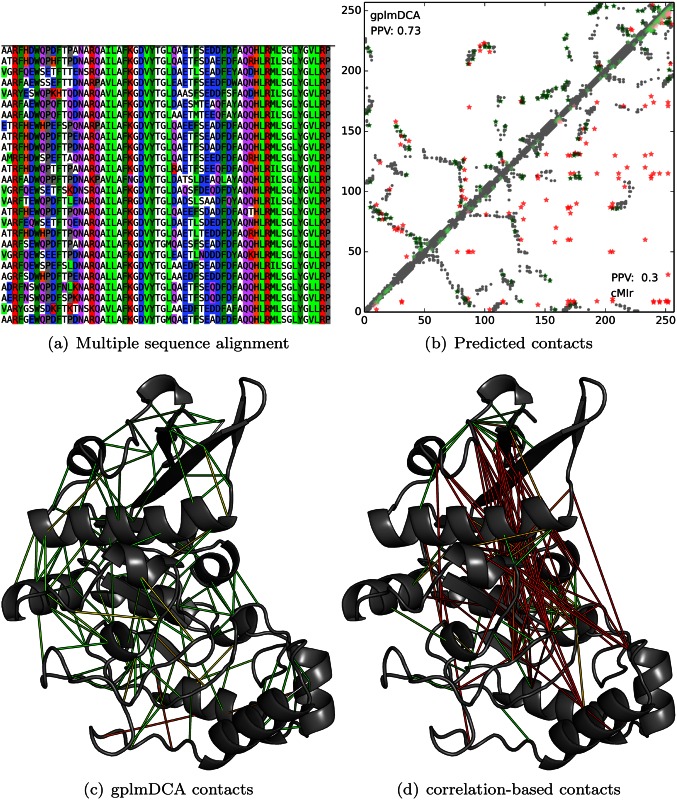
CASP11 free modelling target T0806, YaaA protein of *Escherichia coli*, for which contact prediction has led to accurate predictions of structure without relying on template information. Panel (A) depicts a fragment of multiple sequence alignment used in shown predictions (residues 60–120, with some very similar sequences removed for the sake of clarity). Panel (B) shows a plot of top L/2 contacts predicted by gplmDCA (upper left corner) and correlations-based mutual information method, with alignment filtered for columns and rows containing too many gaps and corrected for phylogenetic bias (Dunn et al., 2008). Panels (C) and (D) depict the predicted contacts plotted against the experimentally determined protein structure, color-coded for distance (green—contacting in real structure; red—noncontacting).

The core of the DCA is to score amino acid pairs by the strength of the interaction matrices *J*_*ij*_ in Eq 1, illustrated in Fig 1.

To determine such *J*’s from a multiple sequence alignment is in general a nontrivial task. Maximum likelihood is typically not feasible because of the size of the problem, and one has to resort to approximations or to weaker inference criteria such as pseudolikelihood. In addition, the number of parameters is large, the problems are usually undersampled, and thus regularization is necessary. These latter issues have been dealt with extensively in the literature [10,15,16] and will not be considered further here. The fundamental observation that scoring pairs by the *J*_*ij*_’s is a much better predictor of spatial proximity than measures of correlation, such as the *f*_*ij*_, or simpler modeling approaches based, e.g., on sequence profiles.

## The Elementary Counterargument

By the logic of the DCA procedure itself, maximum entropy provides no grounds to believe in Eq 1. The DCA starts from the knowledge of the whole multiple sequence alignment and not only from *f*_*i*_(*k*) and *f*_*ij*_(*k*,*l*). Therefore, Eq 1 is not the most unbiased representation of our knowledge of the system. It is a representation of the subset of our knowledge about the system, which remains after the data have first been compressed from the whole multiple sequence alignment to *f*_*i*_(*k*) and *f*_*ij*_(*k*,*l*). Though elementary, this argument is decisive. However, maximum entropy also has problems independently of DCA, as I will now show.

## On Learning a Model from Data That Were or Were Not Generated by the Same Model

Whether or not the data compression as above means loss of information depends on the data and how that were generated. First, consider the case favorable to maximum entropy, when the data actually were generated by Eq 1. It is well known that empirical averages of the conjugate quantities, i.e., *f*_*i*_(*k*) and *f*_*ij*_(*k*,*l*), are then a set of sufficient statistics; the inference of Potts model parameters can then in theory be done equally well from *f*_*i*_(*k*) and *f*_*ij*_(*k*,*l*) as from the whole data set. However, in practice, inferring model parameters in Eq 1 from *f*_*i*_(*k*) and *f*_*ij*_(*k*,*l*) is computationally hard and cannot be done exactly for large enough systems. All methods that attempt to do so rely on approximations [10]—for instance, variational approximations [17]—which lead to estimators that are not statistically consistent. The other class of approximate inference methods widely used in DCA, known as pseudolikelihood and which do lead to consistent estimators, instead keep all the data and never compress to *f*_*i*_(*k*) and *f*_*ij*_(*k*,*l*). In this context, it has recently been rigorously shown that reducing data to sufficient statistics for the task of inferring large models in an exponential family is very suboptimal [18], partially reversing the traditional statistics view of these problems.

Now, consider the more natural case that the data in fact were generated by another probabilistic model, such as an exponential model including both second- and third-order interactions, or a mixture model. Given enough data, the sample frequencies *f*_*i*_(*k*) and *f*_*ij*_(*k*,*l*) will then converge to their ensemble values, and Eq 1 can be used to determine interaction coefficients *J*’s. It is obvious that these inferred *J*’s generally cannot be identified with parameters in the model used to generate the data. For instance, an exponential model including only linear and third-order interactions will typically give rise to nontrivial pair frequencies *f*_*ij*_(*k*,*l*) yielding nontrivial *J*’s using Eq 1, yet the generative model then (by assumption) has no pairwise interactions at all. It is less evident that the relationship of these inferred *J*’s to the model generating the data can be described precisely. As these results from the branch of statistics known as information geometry are not as well known as they perhaps deserve to be, I give a brief summary: the Potts model family can be seen as a subfamily of all probability distributions on *N* variables, and choosing a Potts model with the same first and second moments as the data is a projection, called an *m*-projection. The Potts model family can also be considered a submanifold of the manifold of all probability distributions on *N* variables with the Fisher information matrix as metric, and the result of the *m*-projection can be described in two ways [19]. The first is variational: the result is the Potts model closest to the distribution, generating the data in the sense of minimizing KL divergence. The second is geometric: the result is also the normal intersection of the Pott model submanifold, with a geodesic that in the larger manifold of all probability distributions passes through the distribution that generated the data. In [19], the interested reader may find the details of how this geodesic is determined, which explains what is meant by “geometry” in information geometry. The central fact, entirely in agreement with common sense, is that unless the data actually was generated from Eq 1, the remaining error is finite.

An example of this effect has appeared in DCA. Standard multiple sequence alignments cannot exactly be generated by Eq 1 since alignments contain stretches of the gap variable that represent, in the tabular form, the effects of insertions and deletions. Such sequences are very unlikely to occur in independent draws from Eq 1 in the same manner as long strings of sixes are very unlikely when throwing a fair dice. Prediction performance has been shown to be improved by modifying Eq 1 to contain penalty terms for such stretches of gaps [20]. While these modified Potts models can be construed as maximum entropy models constrained both by pairwise frequencies and frequencies of stretches of gaps, they do illustrate the potential advantage of retaining more information available in the data.

Furthermore, a multiple sequence alignment manifestly contains information on secondary structure and solvent accessibility, which cannot so far be reliably deduced from Eq 1 but which is well predicted by widely adopted software packages, such as NetSurfP [21]. This information forms priors on possible spatial contacts of the type: if residues *i* and *j* are in spatial contact and are both in *α*-helices, then it is very unlikely that residues *i* + 2 and *j* + 2 are also in spatial contact. Indeed, the currently best-performing DCA methods include such prior information, albeit typically not in a very explicit manner [22,23]. These methods are hence quite far from a maximum entropy approach, as Jaynes’ conditional “subject to whatever is known” would then be specified by a one (or several) computer programs, and not by a finite set of constraints.

## Maximum Entropy in Statistical Physics

The conceptual appeal of the maximum entropy argument is that it immediately leads to the Boltzmann distribution of equilibrium statistical physics. However, unless it is assumed that the effects of mutation, selection, and genetic drift in a sufficiently large domain of life are well described by a process obeying detailed balance, the proper analogue must be to the nonequilibrium statistical physics. For a review providing a dictionary between models in statistical physics and models in population genetics, see [24]; for more advanced aspects, see [25,26].

When Jaynes introduced the maximum entropy approach, comparatively little was known in nonequilibrium statistical physics, and maximum entropy could have been envisaged a viable approach. This situation has changed in the almost 60 years that have intervened, and it is now settled that this was not the case. The problem stems from the fact that nonequilibrium systems with a flux exhibit long-range correlations [27,28]. In a physical system, the flux could be of heat from a hotter to a colder boundary, of particles from a source to a sink, or of another quantity that is neither produced nor destroyed in the interior of the system. In Boltzmann distributions built on simple energy functions, long-range correlations only appear at critical points and generally therefore disappear if a system parameter is varied. While there is a substantial literature of putative criticality of nonequilibrium systems, no convincing general mechanism has ever been found. To the contrary, in precise mathematical models known as simple symmetric exclusion processes (SSEP), it has over the last 15 years been shown that the long-range correlations are but consequences of long-range effective interactions [29,30]. That is, the stationary probability distributions in SSEP can be written in exponential form

$$P(a_{1},\ldots ,a_{N})=\frac{1}{Z}\text{exp}\left(V(a_{1},\ldots ,a_{N})\right)$$ (2)

but with a quite complicated function *V*. In fact, *V* contains pairwise interactions at arbitrary spatial separations as well as higher-order interactions of all orders and all spatial separations (though all, miraculously enough, explicitly known). To arrive at such distributions through maximum entropy, one would need an exceedingly large number of constraints of different types. Maximum entropy is therefore not viable as a physical theory because the conditional “subject to whatever is known” encompasses so much that the maximization is in practice an empty concept.

## The Problem

The success of DCAs, which typically try to infer models with hundreds of thousands of parameters from thousands to tens of thousands of examples, can be phrased as the maxim “it is useful to learn exponential models of Big Data.” Why is this so? Let us emphasize that in DCA, the validation of Eq 1 is done with information other than sequence information (protein structures), and hence the connection between statistical models (i.e., the matrices *J*_*ij*_) and the evaluation criterion is indirect, which in principle makes the success of DCAs even more surprising.

Apologetics for maximum entropy is not seldom based on the subjective view of probability, indeed also used by Jaynes [11,12]. This does not work for the DCA by the elementary counter-argument given above. However, on a similar note, it could be that the DCA is but an adequate predictor in the present situation, in which data is severely undersampled compared to the models being learned. Against this possibility speaks the well-known fact that it is not an easy task to improve on DCA, and while the current direction in the literature indeed goes in this direction of more data-driven approaches [22,23], the resulting prediction improvements are hard to disentangle from the use of other information. Moreover, at the current pace of sequencing, we will for many protein families for DCA soon be in the classical setting of statistics where the number of observations is larger than the number of parameters, hence this possibility (if it applies today) could soon be moot.

A second and more intriguing possibility is that naturally occurring probabilities actually do tend to take the exponential form (Eq 2), in analogy with large deviation theory in probability and the Boltzmann distribution of equilibrium statistical mechanics in particular. In fact, there is, as already discussed, a close parallelism between the mathematical models of population genetics and those of statistical physics. For example, an interesting recent contribution shows that for the “house of cards” model of population genetics, and in the successional mutation regime, detailed balance (in genotype space) indeed does hold for long time scales [31]. If such an explanation would turn out to work, it would say something important about the action of evolution on large scales, which would be interesting by itself. It would also be based on the objective view of probability and position DCA within mainstream statistical inference. In conclusion, while there is ample reason to be excited about the applications and prospects of DCA, the foundational question of why it works at all may turn out to be even more fascinating and fruitful in this age of biological Big Data.
